# Leveraging existing 16S rRNA microbial data to identify diagnostic biomarker in Chinese patients with gastric cancer: a systematic meta-analysis

**DOI:** 10.1128/msystems.00747-23

**Published:** 2023-10-03

**Authors:** Jijun Chen, Siru Nie, Xunan Qiu, Shuwen Zheng, Chuxuan Ni, Yuan Yuan, Yuehua Gong

**Affiliations:** 1 Tumor Etiology and Screening Department of Cancer Institute and General Surgery, The First Hospital of China Medical University, Shenyang, Liaoning, China; 2 Key Laboratory of Cancer Etiology and Prevention, Liaoning Education Department, The First Hospital of China Medical University, Shenyang, Liaoning, China; 3 Key Laboratory of GI Cancer Etiology and Prevention, The First Hospital of China Medical University, Shenyang, Liaoning, China; Universita degli Studi di Trento, Trento, Italy

**Keywords:** 16S rRNA, diagnostic biomarker, gastric cancer, meta-analysis

## Abstract

Gastric cancer is the second most prevalent and deadly cancer in China. Microbiota play an important role in gastric tumorigenesis. However, the available microbial marker studies for gastric cancer do not have consistent results. We searched PubMed for 16S rRNA sequencing in relevant literature on Chinese patients from 1 January 2005 to 18 July 2022, and 16 original articles were finally obtained. Alpha diversity, beta diversity, and bacterial taxa were used to explore the differences in gastric microbiota. Linear discriminant analysis of effect size and a random forest model were used to find the combination of genera with the best diagnostic efficacy. *Streptococcus*, *Pseudomonas*, *Fusobacterium*, *Selenomonas*, *Peptostreptococcus*, and *Prevotella* showed significant differences between gastric cancer and non-gastric cancer, but a single genus performed poorly in identifying patients with gastric cancer. However, a combination of genera *Streptococcus*, *Peptostreptococcus*, *Selenomonas*, *Pseudomonas*, and *Prevotella* had excellent performance in screening gastric cancer with the median area under the curve values of 0.7525 (range: 0.5859–0.9350), 0.8818 (range: 0.7397–0.9533), and 0.7435 (range: 0.7131–0.8483) in the Matched, Unmatched, and Other groups, respectively. Therefore, the results indicated that this combination of genera has good diagnostic efficacy and wide applicability for patients with gastric cancer, which may provide new clues for the non-invasive diagnosis of gastric cancer.

**Importance:**

Gastric cancer is a significant and growing health problem in China. Studies have revealed significant differences in gastric microbiota between patients with gastric cancer and non-cancerous patients, suggesting that microbiota may play a role in tumorigenesis. In this meta-analysis, existing 16S rRNA microbial data were analyzed to find combinations consisting of five genera, which had good efficacy in distinguishing gastric cancer from non-cancerous patients in multiple types of samples. These results lend support to the use of microbial markers in detecting gastric cancer. Moreover, these biomarkers are plausible candidates for further mechanistic research into the role of the microbiota in tumorigenesis.

## INTRODUCTION

Gastric cancer (GC) remains an important cancer globally, ranking fifth in incidence and fourth in mortality. It is about twice as common in men as in women (1). East and Southeast Asia have some of the highest incidences of GC in the world, representing more than 60% of all GC cases in 2018 (2). In China, GC is the second most prevalent and second most deadly cancer, with mortality/morbidity (0.845) and 5-year prevalence (27.6/100,000) higher than in most developed countries (3). Since the discovery of *Helicobacter pylori* (*Hp*), the stomach is no longer considered a sterile environment and is thought to contain a complex ecosystem of multiple microorganisms. Microbial factors have been recognized as risk factors for GC and have received more attention recently. Studies have shown that microorganisms in the upper gastrointestinal tract can promote cancer by promoting inflammation (4) or by interacting with pathogens (5). Therefore, the exploration of microbiota-based GC diagnostic markers in the Chinese population is of great value for the identification, prevention, and treatment of GC.

16S rRNA is a component of the ribosomal 30S subunit that is highly conservative and specific in prokaryotes. Sequencing the variable region of 16S rRNA is currently the most common method for studying microorganism diversity. The use of 16S rRNA amplicon sequencing showed differences among the phyla *Proteobacteria* and *Firmicutes*, and the genera *Streptococcus*, *Prevotella*, and *Fusobacterium* in cancerous and adjacent normal tissues of GC (6, 7). However, some studies have reported regional differences in microbiota profiles, and the microorganisms responsible for GC may not be the same in different regions. A recent study including 110 Mongolian and 83 Han Chinese stool samples from healthy individuals showed that Mongolians have a more unique and diverse gut microbial community than Han Chinese, suggesting that the environment influences the microbial community (8). Nevertheless, there were significant differences in gastric mucosal microbial communities between patients with GC and healthy people in different studies from the Chinese population. For example, a study conducted in Xi'an and Inner Mongolia identified a combination of several operational taxonomic units (OTUs)—Peptostreptococcus_OTU16, Parvinomonas_OTU35, Streptococcus_OTU68, Dialister_OTU151, and Slackia_OTU174—with good results in diagnosing GC in gastric mucosal samples (9). In addition, a multicenter study in China, pooling samples from Beijing, Tianjin, Nanjing, Shanghai, Xiamen, and Guangzhou, found that the combination of the species *Streptococcus anginosus* and *Streptococcus constellatus* had high sensitivity and diagnostic efficacy in identifying patients with GC and could be used for GC screening (10). The differences in the results of the above studies based on Chinese populations may be explained by differences in sample sizes, controls, types, and the selection of sequencing platforms and variable regions among studies. This highlights the need for systematic analysis of microbial diagnostic markers for GC.

Based on this, we performed a meta-analysis using the existing 16S rRNA sequencing data published in previous articles to find microbial markers with diagnostic value for GC in the Chinese population. This study is of great significance for exploring microbial markers with a clinical diagnostic role for GC.

## MATERIALS AND METHODS

### Public data collection

**Fig 1:**
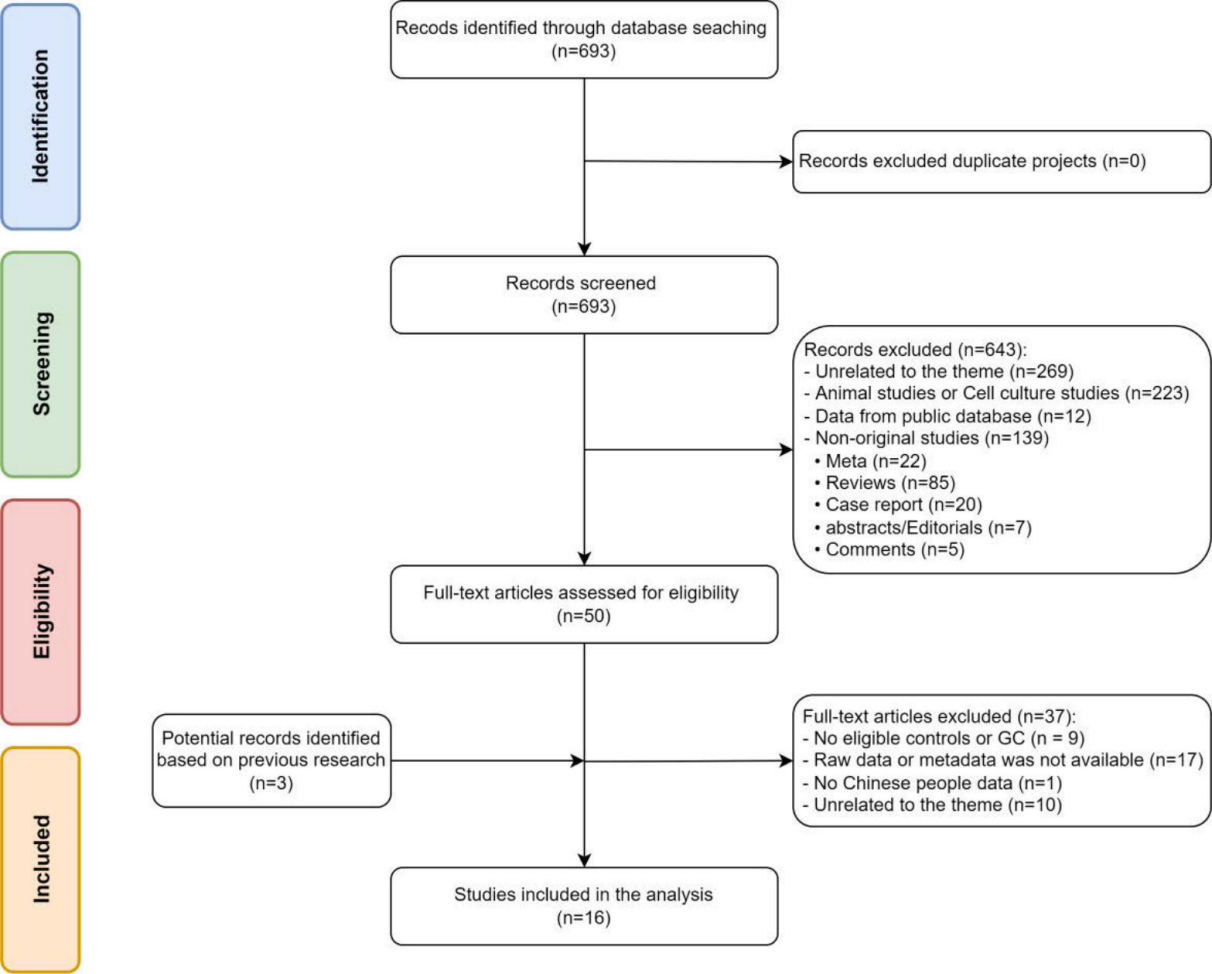
Study flow chart.

We searched PubMed for 16S rRNA sequencing relevant literature of Chinese patients from 1 January 2005 to 18 July 2022 with the following search strategy: ((((microbiome OR microbial OR microbiota [MeSH Terms]) OR microflora OR bacterial OR dysbiosis) AND (gastric [MeSH Terms] OR stomach OR upper digestive tract OR upper gastrointestinal tract)) AND ((lesion OR cancer [MeSH Terms] OR neoplasia OR neoplasms OR malignancy OR tumor OR carcinoma OR adenocarcinoma OR premalignancy OR premalignant OR tumorigenesis OR carcinogenesis) OR intestinal metaplasia OR gastritis)) AND ((Chinese) OR (China)). Data were included according to the following criteria: the study population was a Chinese population containing GC and NGC [Non-GC, including distal normal control (NC), healthy control (HC), superficial gastritis (SG), and chronic gastritis (CG)], providing publicly available sequence and subgroup information. A total of 693 articles was obtained using the above search criteria, and after reviewing the titles and abstracts, 643 irrelevant articles were excluded. Among the remaining 50 articles, 37 irrelevant articles were excluded by reading the full text. In addition, three relevant studies were added manually by literature reading, and so 16 original articles were finally included (Fig. 1).

### Data preprocessing

Raw sequence data and metadata were obtained from Sequence Read Archive (SRA; https://www.ncbi.nlm.nih.gov/sra) and European Nucleotide Archive (ENA; https://www.ebi.ac.uk/ena/browser/home). Quality checks were performed using FastQC and each data set was processed separately using QIIME2 (version 2021.2) (11). Single- or double-ended FASTQ files were imported to generate QIIME2 files. The double-ended data were merged using the q2-vsearch (12) plugin, and then the quality filter plugin was used to quality filter the merged or single-ended data, setting a minimum quality score of 20. Next, the connected and quality-filtered sequences were noise reduced using the Deblur noise reduction program (13) to obtain single sequence variants. Then we used the default parameters to annotate species by QIIME2 using a well-trained plain Bayesian classifier (99% similarity) (https://github.com/QIIME2/q2-feature-classifier) (14) using Greengenes full-length reference sequences. Any sequences identified as members of the eukaryotic, archaeal, mitochondrial, chloroplast, and cyanobacterial lineages were removed.

### Community analysis

To elucidate species diversity between the NGC and GC, we performed alpha-diversity analysis using the QIIME2 platform. After excluding samples with <1,000 reads, community richness, diversity, and evenness were expressed by observed features, Shannon index, and evenness index, respectively. In each data set, we used a rank transformation to ensure that the data obeyed a normal distribution. For the transformed data, we tested the hypothesis that the alpha diversity significantly differed between the NGC and GC groups using a linear mixed-effects model. We plotted sparse curves at the median sequencing depth for each data set to determine the stability of the alpha diversity results. We measured differences between individuals by calculating beta diversity (BrayCurtis distance, weighted unifrac distance, and unweighted unifrac distance) and used permutational multivariate ANOVA (PERMANOVA) to determine whether there were significant differences between NGC and GC patients.

### Linear discriminant analysis of effect size

We used linear discriminant analysis of effect size (LEfSe) (15) to assess how the microbiome varied with disease state. This determines the features most likely to explain differences between classes by coupling standard tests for statistical significance with additional tests encoding biological consistency and effect relevance. We used the Galaxy implementation of LEfSe (accessed on 20 March 2022; available at http://huttenhower.sph.harvard.edu/galaxy) with default options. Differences were evaluated by differentiating the threshold of the log-linear discriminant analysis (LDA) score of the features, which was set to 2.0.

### Random forest classification analysis

Incorporating the common differential genera obtained from the LEfSe. we constructed a random forest model using the R package randomForest (16). Each data set was randomly divided 7:3 into training and test data sets. For all models, ntree was set to 500, and mtry was set to the square root of the number of taxa within the model. The mean decline in accuracy (MDA) is a measure of the importance of each categorical unit to the overall model, and we obtained the overall importance ranking by normalizing the MDA values of the categorical units in each model (Z-transformation).

### Statistical analysis

SPSS version 22.0 (SPSS Inc., Chicago, IL, USA) was used for statistical analysis. GraphPad Prism 8.0 (GraphPad Software Inc., San Diego, CA, USA) was used for graphing. Mann-Whitney U test was used to check the alpha diversity parameter. Differences in colony structure between groups were calculated using Bray-Curtis distances, weighted unifrac distances, and unweighted unifrac distances, and PERMANOVA was performed using the Vegan package (v2.5.7) in R (version 4.1.2) to detect variability in beta diversity between groups. The LEfSe algorithm was used to identify specific microbial taxa and functions that differed significantly between groups. Differences with absolute LDA scores >2.0 were considered significant. Random forest models were constructed using the randomForest package (v4.6.14). The ROC curves were plotted using the pROC (1.18.0) and ggplot2 (3.3.6) packages.

## RESULTS

### Basic characteristics of the included data set

The 16 original articles have differences in DNA extraction, PCR primer, Sequence Region, and Sequence platform. The main methods used for DNA extraction are the QIAamp DNA Mini Kit and the QIAGEN DNeasy Kit. The most commonly used PCR primers are 515F/806R, followed by 338F/806R, 319F/806R, etc. In terms of Sequence Region, Regions V3-V4 and V4 are the most common, while the Illumina platform is used for sequencing, with Illumina MiSeq being the most common (Tables 1 to 3).

**TABLE 1:** Characteristics of the studies included in the Matched group

ID	Study	Geographical location	DNA extraction	PCR primer	Seq region	Seq plat^*g*^	Samples	Source of data	Sample type	Data included	Data availability (accession no.)
	Tseng et al. (17)	Taizhong, Taiwan	QIAGEN DNA Mini Kit	27F^*e*^/534R^*f*^	V1–V3^*h*^	Illumina MiSeq	GC—6,^*a*^ NC—6,^*b*^ total—12	SRA^*d*^	Gastric mucosa	×	PRJNA282779
S1	Yu et al. (18)	Taiyuan, Shanxi	QIAamp DNA Mini Kit	NA	V3–V4	Illumina MiSeq	GC—80, NC—77, total—157	SRA	Gastric mucosa	√	PRJNA310127
S2	Liu et al,2018	Hangzhou, Zhejiang	QIAamp DNA Mini Kit	319F/806R	V3	Illumina MiSeq	GC—229, NC—230, total—459	SRA	Gastric mucosa	√	PRJNA428883
S3	Coker et al. (9)	Hohhot, Inner Mongolia	QIAamp DNA Mini Kit	515F/806R	V4	Illumina MiSeq	GC—19, NC—17, total—36	SRA	Gastric mucosa	√	PRJNA375772
S4	Coker et al. (9)	Xi'an, Shanxi	QIAamp DNA Mini Kit	515F/806R	V4	Illumina MiSeq	GC—20, NC—20, Total—40	SRA	Gastric mucosa	√	PRJNA375772
S5	Liu et al. (19)	Hangzhou, Zhejiang	QIAamp DNA Mini Kit	319F/806R	V3–V4	Illumina Miseq	GC—59, NC—60, total—119	SRA	Gastric mucosa	√	PRJNA508819
S6	Liu et al. (19)	Linxian, Henan	Mobio PowerSoil DNA	515F/806R	V4	Illumina MiniSeq	GC—36, NC—36, total—72	SRA	Gastric mucosa	√	PRJNA561290
S7	Chen et al. (6)	Shenyang, Liaoning	DNA extraction standard pipeline (20)	515F/907R	V4–V5	Illumina Hiseq	GC—62, NC—62, total—124	SRA	Gastric mucosa	√	PRJNA532731
S8	Zhang et al. (21)	Hefei, Anhui	CTAB method	515F/806R	V4	Illumina NovaSeq	GC—33, NC—36, total—69	SRA	Gastric mucosa	√	PRJNA778008
S9	He et al. (22)	Nanchang, Jiangxi	QIAGEN DNeasy Kit	515F/806R	V4	Illumina Miseq	GC—57, NC—56, total—113	SRA	Gastric mucosa	√	PRJNA481413

^*a*^ GC, gastric cancer.

^*b*^ NC, adjacent normal control.

^*c*^ ENA, European Nucleotide Archive.

^*d*^ SRA, Sequence Read Archive.

^*e*^ F, forward (5′-3′) direction.

^*f*^ R, reverse (3′-5′) direction.

^*g*^ seq plat, sequencing platform.

^*h*^ V1, V3, V4, and V5 are variable regions of the 16S rRNA gene.

**TABLE 2:** Characteristics of the studies included in the Unmatched group^*c*^

ID	Study	Geographical location	DNA extraction	PCR primer	Seq region	Seq plat	Samples	Source of data	Sample type	Data included	Data availability (accession no.)
	Wang et al. (23)	Qingdao, Shandong	QIAGEN DNeasy Blood and Tissue Kit	8F^*g*^/533R^*h*^	V1–V3^*i*^	454GS-FLX	GC—6,^*a*^ CG—6,^*b*^ total—12	SRA^*f*^	Gastric mucosa	×	PRJNA289186
S10	Coker et al. (9)	Hohhot, Inner Mongolia	QIAamp DNA Mini Kit	515F/806R	V4	Illumina MiSeq	GC—19, SG—55,^*c*^ total—74	SRA	Gastric mucosa	√	PRJNA375772
S11	Coker et al. (9)	Xi'an, Shanxi	QIAamp DNA Mini Kit	515F/806R	V4	Illumina MiSeq	GC—20, SG—54, total—74	SRA	Gastric mucosa	√	PRJNA375772
S12	Wang et al. (24)	Beijing	QIAamp DNA	515F/806R	V4	Illumina MiSeq	GC—29, HC—30,^*d*^ CG—21, total—80	ENA^*e*^	Gastric mucosa	√	PRJEB26931
S13	Wang et al. (24)	Qingdao, Shandong	QIAGEN DNeasy Tissue Kit	338F/806R	V3–V4	Illumina HiSeq	GC—60, CG—30, total—90	SRA	Gastric mucosa	√	PRJNA313391
S14	Zhang et al. (25)	Nanjing, Jiangsu	E.Z.N.A Stool DNA Kit	338F/806R	V3–V4	Illumina MiSeq PE300	GC—15, SG—17, total—32	SRA	Gastric mucosa	√	PRJNA634837
S15	Li et al. (26)	Changchun, Jilin	NA	341F/806R	V3–V4	Illumina MiSeq PE250	GC—15, CG—21, total—36	SRA	Gastric mucosa	√	PRJNA634837
S16	He et al. (22)	Nanchang, Jiangxi	QIAGEN DNeasy Kit	515F/806R	V4	Illumina Miseq	GC—57, SG—114, total—171	SRA	Gastric mucosa	√	PRJNA481413

^*a*^ GC, gastric cancer.

^*b*^ CG, chronic gastritis.

^*c*^ SG, superficial gastritis.

^*d*^ HC, healthy control.

^*e*^ ENA, European Nucleotide Archive.

^*f*^ SRA, Sequence Read Archive.

^*g*^ F, forward (5′-3′) direction.

^*h*^ R, reverse (3′-5′) direction; seq plat, sequencing platform.

^*i*^ V1, V3, V4 are variable regions of the 16S rRNA gene.

**TABLE 3:** Characteristics of the studies included in the Other group

ID	Study	Geographical location	DNA extraction	PCR primer	Seq region	Seq plat^*g*^	Samples	Source of data	Sample type	Data included	Data availability (accession no.)
S17	Liu et al. (19)	Linxian, Henan	Mobio PowerSoil DNA	515F^*e*^/806R^*f*^	V4	Illumina MiniSeq	GC—36,^*a*^ HC—36,^*c*^ total-72	SRA^*d*^	Gastric swab sample	√	PRJNA561290
S18	Xu et al, 2020	Hefei, Anhui	E.Z.N.A. Soil DNA Kit	341F/806R	V3–V4^*h*^	Illumina MiSeq	GC—181, HC—112, total—293	SRA	Tongue coating	√	PRJNA639445
S19	Zhang et al. (21)	Hefei, Anhui	CTAB method	515F/806R	V4	Illumina NovaSeq	GC—70, HC—70, total—140	SRA	Oral swab sample	√	PRJNA778008
S20	Zhang et al. (21)	Hefei, Anhui	CTAB method	515F/806R	V4	Illumina NovaSeq	GC—70, HC—49, total—119	SRA	Stool	√	PRJNA778008
S21	Qi et al. (27)	Taiyuan, Shanxi	E.Z.N.A. Stool DNA Kit	341F/806R	V3–V4	Illumina Miseq	GC—116, HC—88, total—204	SRA	Stool	√	PRJNA478252
S22	He et al. (22)	Nanchang, Jiangxi	QIAGEN DNeasy Kit	515F/806R	V4	Illumina Miseq	GC—28, SG—42,^*b*^ total—70	SRA	Gastric fluid	√	PRJNA481413

^*a*^ GC, gastric cancer.

^*b*^ SG, superficial gastritis.

^*c*^ HC, healthy control.

^*d*^ SRA, Sequence Read Archive.

^*e*^ F, forward (5′-3′) direction.

^*f*^ R, reverse (3′-5′) direction.

^*g*^ seq plat, sequencing platform.

^*h*^ V3 and V4 variable regions of the 16S rRNA gene.

We divided samples into three groups based on whether they originated from the same individual and sample type: Matched, Unmatched, and Other groups. A total of 10 gastric mucosal microbiome data were included in the Matched group. Of these, Tseng et al. (17) were excluded due to a small sample size and ultimately included nine data from eight studies (S1–S9): Yu et al. (18), Liu et al. (28), Coker et al. (9), Ling et al. (19), Liu et al. (29), Chen et al. (6), Zhang et al. (21), and He et al. (22). A total of 1,189 tissue samples were included in this group, of which 595 were GC samples and 594 were NGC samples (Table 1). A total of eight gastric mucosal microbiome studies were included in the Unmatched group. Of these, Wang et al. (30) were excluded due to small sample size and ultimately included seven data from six studies (S10–S16): Coker et al. (9), Wang et al. (24), Wang et al. (31), Zhang et al. (25), Li et al. (26), and He et al. (22). A total of 557 tissue samples were included in this group: 215 were GC samples and 342 were NGC samples (Table 2). The Other group included six data from five studies (S17–S22) and included microbiome data from gastric mucosal swab, oral swab, tongue coating, stool, and gastric fluid: Liu et al. (29), Xu et al. (32), Zhang et al. (21), Qi et al. (27), and He et al. (22). A total of 898 samples were included in this group: 501 were GC samples and 397 were NGC (Table 3).

### Community analysis showed significant differences in microbiota between GC and NGC

**Fig 2:**
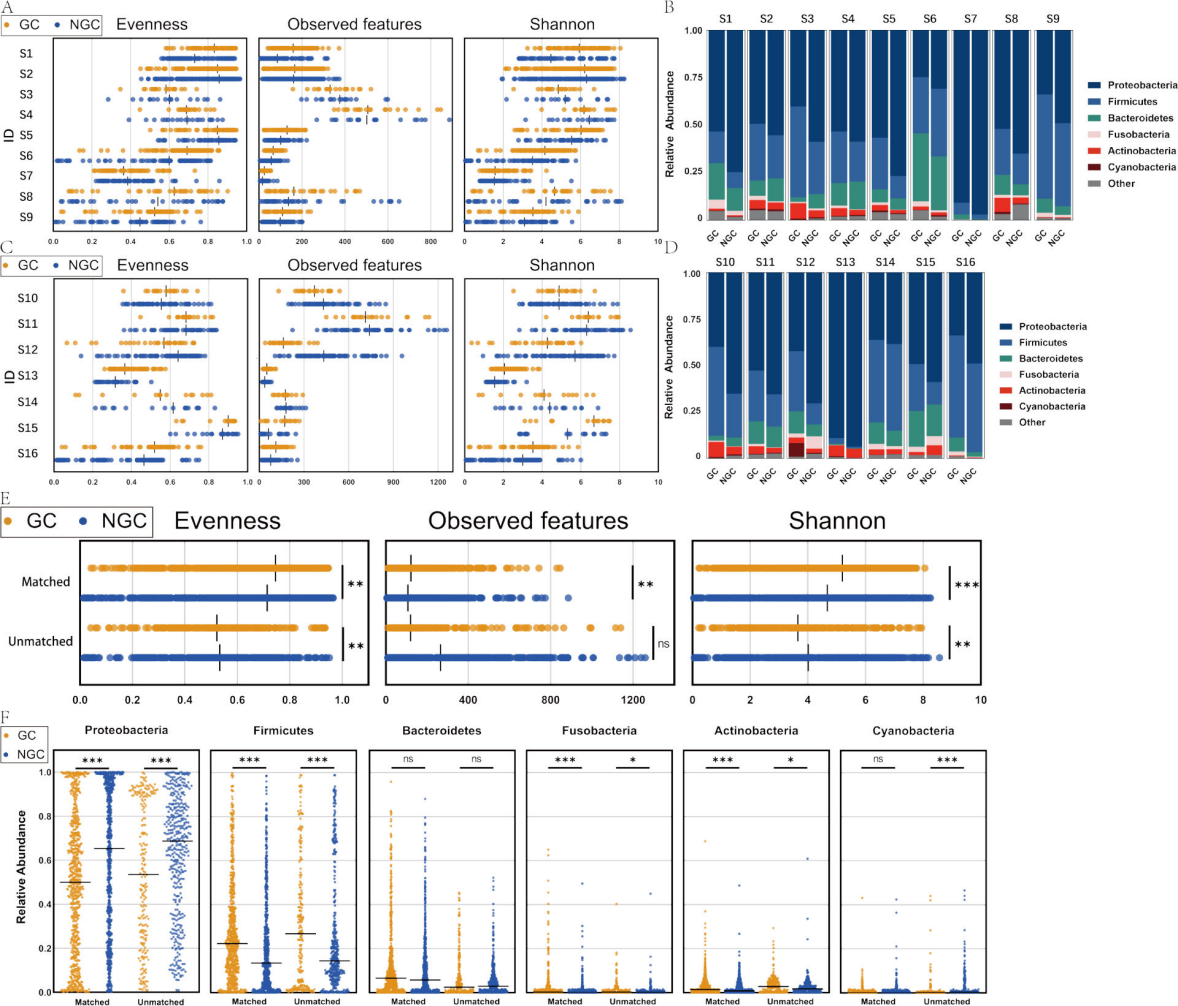
Community analysis between GC and NGC in the Matched and Unmatched groups. (**A**) Alpha-diversity indicators evenness index, observed features, and Shannon index in the Matched group of data in each group. (**B**) The microbiota composition of each data set in the Matched group. (**C**) Evenness index, observed features, and Shannon index in the Unmatched group of data in each group. (**D**) The microbiota composition of each data set in the Unmatched group. (**E**) Evenness index, observed features, and Shannon index in the Matched and Unmatched groups after controlling for variables with a linear mixed-effects model. (**F**) After controlling for variables with a linear mixed-effects model, the six most highly expressed phyla in the Matched and Unmatched groups.

To explore the differences in gastric microbiota between GC and NGC, we compared the alpha diversity, beta diversity, and bacterial taxa of each data in the Matched, Unmatched, and Other groups after controlling for differences in study and variable regions. In the Matched group (S1–S9), evenness index, observed features, and Shannon index were significantly higher in the GC group (*P*-values 4.96E−03, 1.12E−04, and 3.27E−07, respectively) (Fig. 2A and E; Table S1). By contrast, in the Unmatched group (S10–S16), evenness and Shannon indexes were significantly lower in the GC group (*P*-value of 0.0074 and 0.0099, respectively), while observed features did not significantly differ between GC and NGC (*P* = 0.9337) (Fig. 2C and E; Table S2). The beta-diversity analysis showed significant differences between GC and NGC in most of the data (Table S4). In terms of bacterial taxa, *Proteobacteria*, *Firmicutes*, and *Bacteroidetes* were the dominant phyla constituting the gastric mucosal microbial community. In addition, the phyla *Cyanobacteria*, *Actinobacteria*, and *Fusobacteria* showed high relative abundance for some data. Compared to the NGC, *Proteobacteria* were significantly lower in the GC group (*P-*value of 4.90E−12 in the Matched and 9.41E−08 in the Unmatched groups), *Firmicutes* were significantly higher in the GC group (*P*-value of 5.44E−09 in the Matched and 2.00E−06 in the Unmatched groups), while *Bacteroidetes* did not differ significantly between the two groups (*P-*value of 9.53E−02 in the Matched and 5.04E−02 in the Unmatched groups) (Fig. 2B, D and F; Table S2). The Other group contained several types of samples, with the bacterial taxa of patients with GC differing significantly between sample types (Fig. S1; Table S3).

### Six genera may have potential diagnostic biomarkers for distinguishing GC from NGC

**Fig 3:**
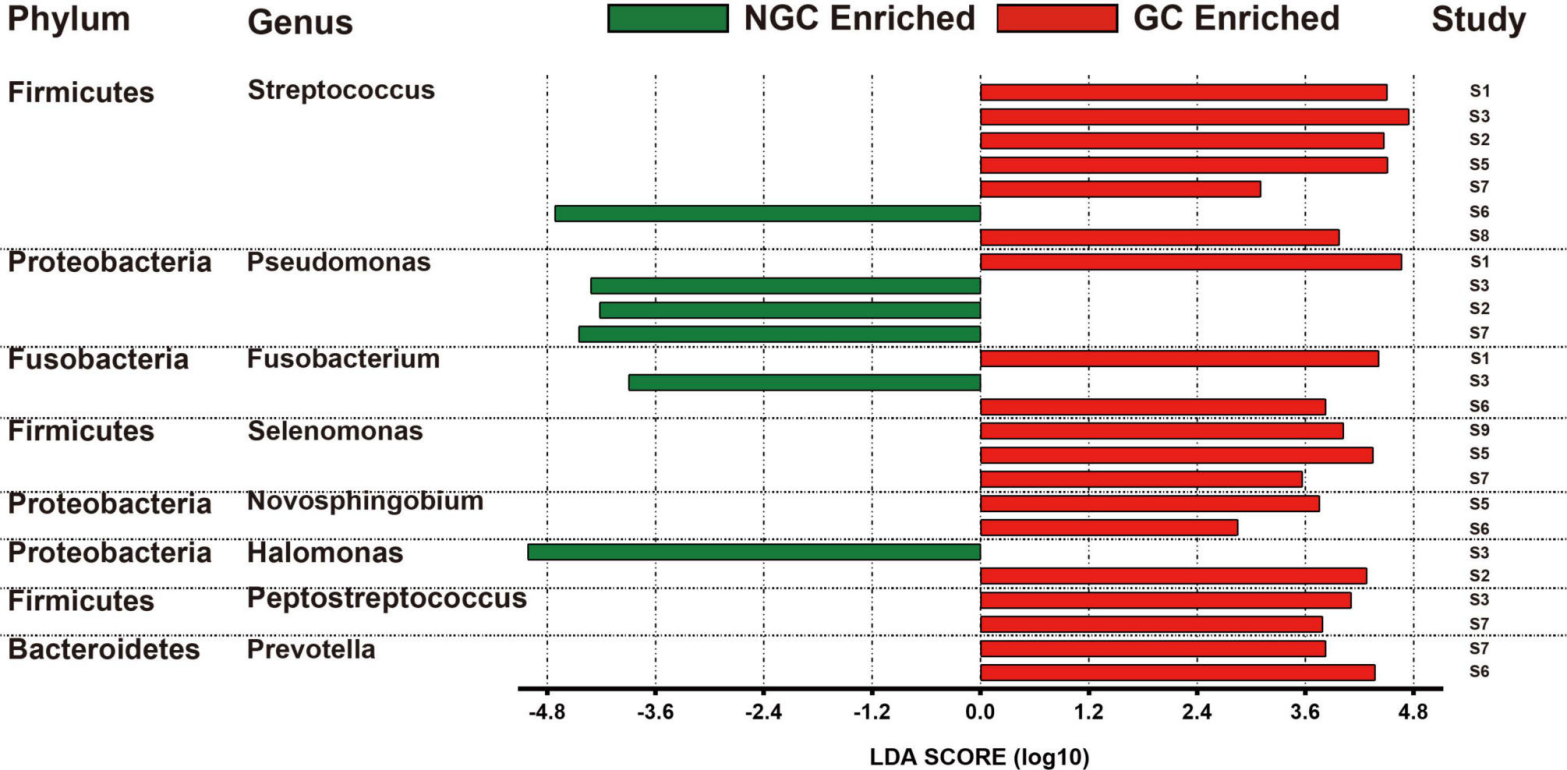
Eight genera obtained by LEfSe.

Bacterial genera with significant differences between GC and NGC should have better efficacy in identifying patients with GC. Therefore, we performed LEfSe on the data from the Matched group to find microbial markers for GC. We summarized the results of LEfSe of data from S1 to S9 groups and selected genera that appeared in two or more data groups. The following eight genera were obtained: *Streptococcus*, *Pseudomonas*, *Fusobacterium*, *Selenomonas*, *Novosphingobium*, *Halomonas*, *Peptostreptococcus*, and *Prevotella* (Fig. 3). However, *Novosphingobium* and *Halomonas* could not be annotated in some of the Matched data, and so were excluded. Finally, six genera *Streptococcus*, *Pseudomonas*, *Fusobacterium*, *Selenomonas*, *Peptostreptococcus*, and *Prevotella* were considered as potential diagnostic biomarkers for distinguishing GC from NGC.

### A single genus not so well-diagnostic biomarkers for distinguishing GC from NGC

**Fig 4:**
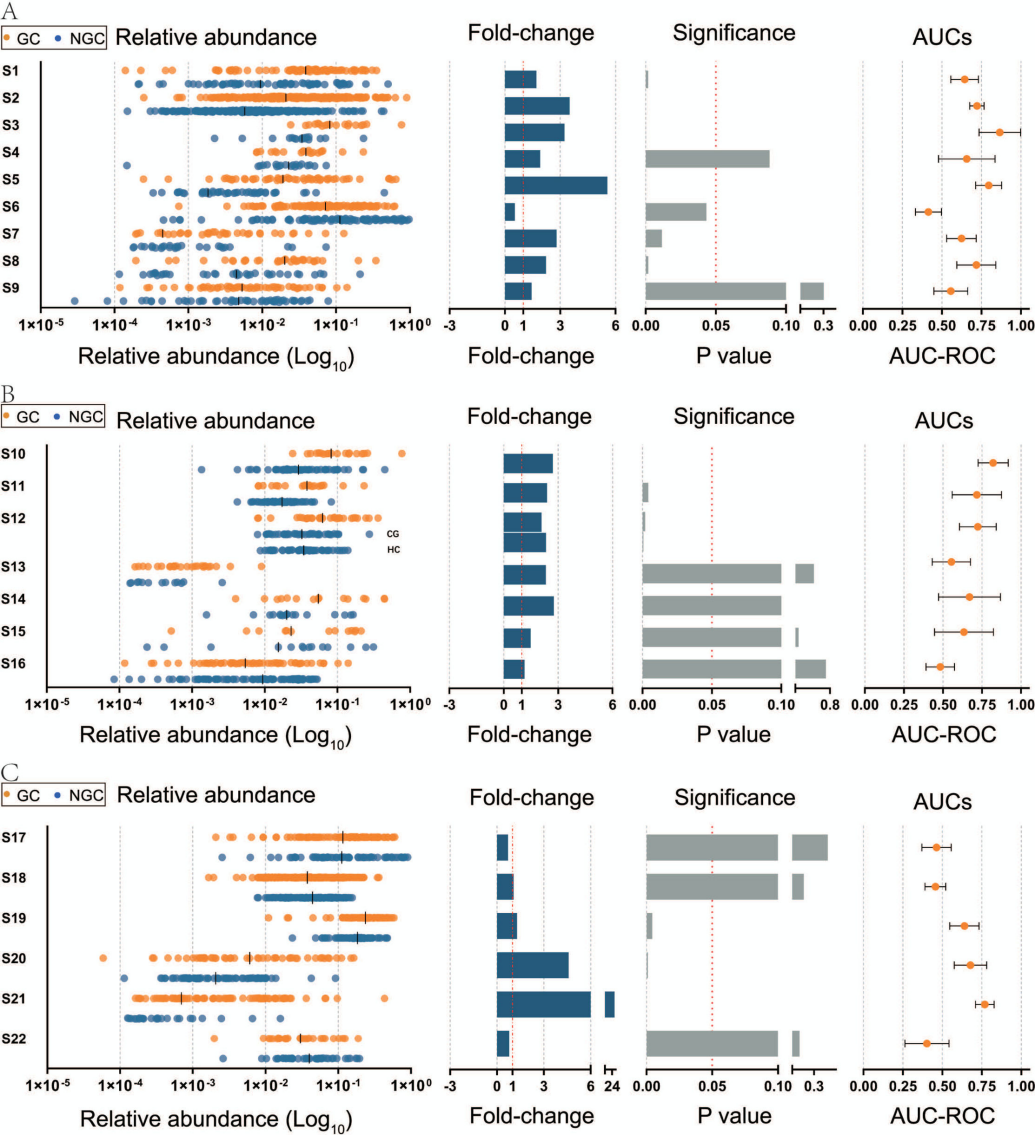
Relative abundance, fold-change, and *P*-value of *Streptococcus* between the GC and NGC in each data set, and AUC of *Streptococcus* as a marker for diagnosis of GC. (**A**) Matched group. (**B**) Unmatched group. (**C**) Other group.

We explored the possibility of each of these six genera alone as diagnostic markers for GC. Given that *Streptococcus* was identified as a potential marker in many data in previous results, we first analyzed its diagnostic ability. In gastric mucosal tissue, the relative abundance of *Streptococcus* was significantly higher in most GC groups than in controls. The median area under the curve (AUC) value was 0.6575 (range: 0.4150–0.8669) in the Matched group (S1–S9) and 0.6706 (range: 0.4831–0.822) in the Unmatched group (S10–S16) (Fig. 4A and B). The results were slightly better in the Unmatched than the Matched groups. In the Other group (S17–S22), the AUCs were 0.6394, 0.6781, and 0.7687 in one group of oral swab samples and two groups of stool samples, respectively, and less than 0.6 in the remaining types of samples (Fig. 4C). However, *Streptococcus* did not show significant differences in the Other group. These results suggested not so well of *Streptococcus* as a diagnostic marker for GC for the majority of data. We supplemented the expression of six genera in all data sets NGC and GC groups, and also analyzed the diagnostic efficacy of the remaining five genera, with results generally similar to those of *Streptococcus*. Details are shown in Fig. S2 and S3.

### The best-performing combination of microbial diagnostic biomarkers obtained by random forest analysis

**Fig 5:**
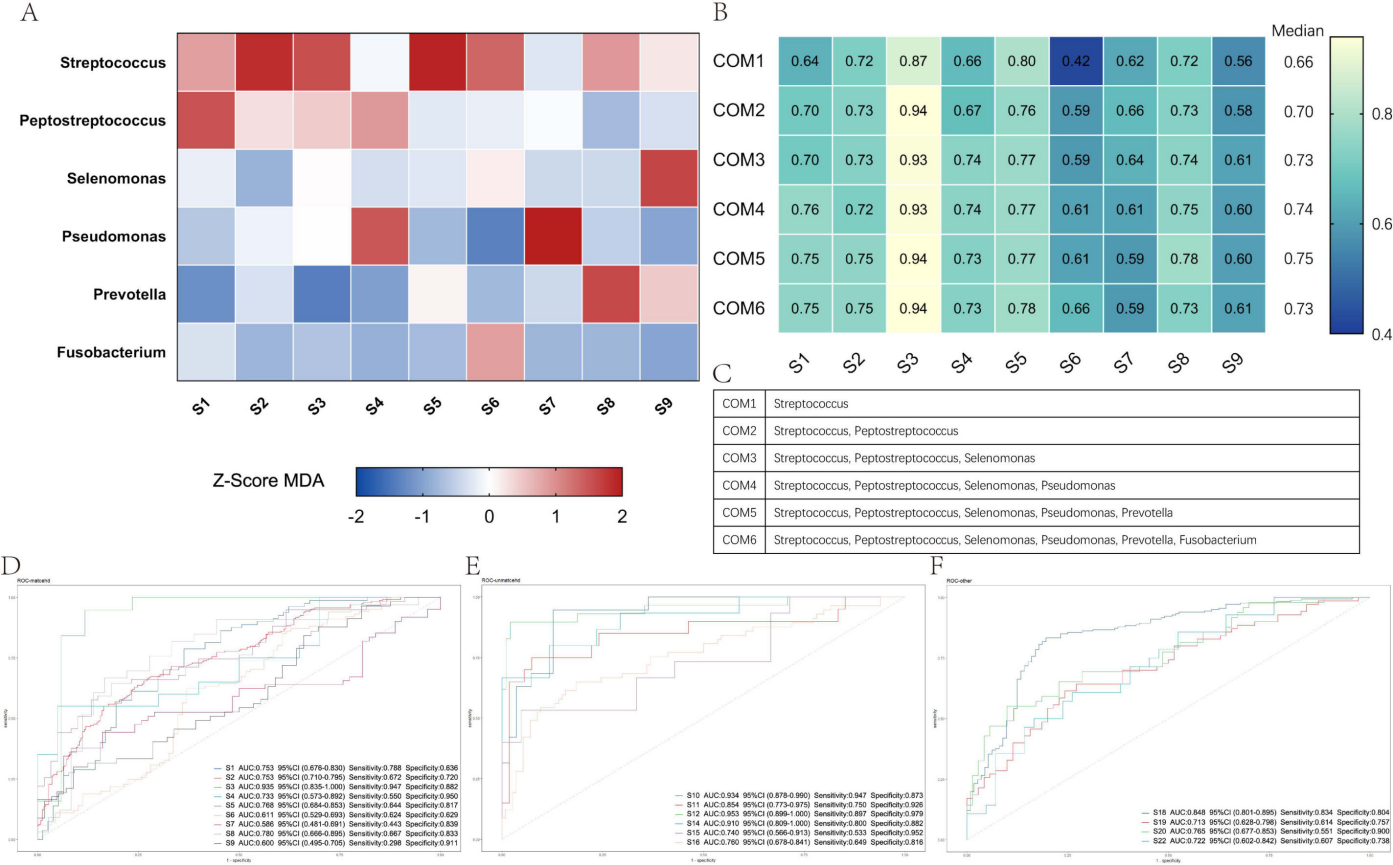
Identifying the best-performing combination of microbial diagnostic biomarkers and validating their diagnostic capabilities. (**A**) Heatmap of MDA values obtained by random forest model after Z-transformation. (**B**) AUC values for each combination in the Matched group for each data set. (**C**) Genus composition in each combination. (**D**) ROC curves of COM5 in the Matched group for each data set. (**E**) ROC curves of COM5 in the Unmatched group for each data set. (**F**) ROC curves of COM5 in the Other group for each data set.

We constructed random forest models in each data set of the Matched group (S1–S9) and Z-transformed the resulting MDA values. The sum of the Z-score MDA results was ranked, and the total importance ranking of each genus in the model was obtained as follows (in order of decreasing importance): *Streptococcus*, *Peptostreptococcus*, *Selenomonas*, *Pseudomonas*, *Prevotella*, and *Fusobacterium* (Fig. 5A). In this model, *Streptococcus* was the most important factor, consistent with our LEfSe results. We obtained six combinations by progressively including genera based on importance ranking and validated the diagnostic efficacy of these combinations in the data set of the Matched group. The results showed that COM5 was the best-performing combination, with a median AUC value of 0.7525 (range: 0.5859–0.9350) (Fig. 5B through D).

### The best-performing combination of microbial diagnostic biomarkers verified in the unmatched and other group

To further explore the ability of the combination in screening patients with GC, we validated the combination COM5 in the Unmatched group (S13 excluded due to lack of *Selenomonas*) and the Other group (S17 excluded due to lack of *Pseudomonas* and S21 excluded due to lack of *Pseudomonas* and *Selenomonas*). Unexpectedly, the combination had an extremely high AUC in the Unmatched group with a median value of 0.8818 (range: 0.7397–0.9533) (Fig. 5E). The AUC was also above 0.7 in all four types of sample data in the Other group, with values of 0.8483, 0.7131, 0.7650, and 0.7219, respectively (Fig. 5F). These results suggested that the bacterial genera that are significantly different from NGC in GC mucosal tissues might also be used in the diagnosis of GC in oral swabs, tongue coating, feces, or gastric juice specimens.

## DISCUSSION

An increasing number of studies have found that microorganisms in the stomach other than *Hp* are closely associated with the development of GC. In this study, we performed a systematic meta-analysis using 16S rRNA sequencing data published in previous articles and identified the best-performing combination of microbial diagnostic biomarkers for distinguishing GC from NGC in Chinese patients. And this biomarker has good diagnostic efficiency in a variety of different types of samples, which has very important clinical value.

We first explored the basic characteristics of the flora in GC versus non-cancerous samples. The alpha diversity indicators (evenness index, Shannon index, and observed features) were elevated in GC from the Matched group, but the evenness and Shannon indexes were decreased in GC from the Unmatched group, after controlling factors. Current studies have not reached a consistent conclusion on the relationship between microbial diversity and gastric disease status, and Dai et al. showed that the diversity and abundance of gastric microbiota were higher in tumor tissues than in non-tumor tissues (7). However, the diversity and richness of peritumoral and tumor tissues were decreased in 276 patients with GC compared with non-tumor tissues (28). Our study may partly explain the inconsistent alpha diversity results in previous studies, which we speculated is related to the controls being from the same individual or different individuals.

LEfSe analysis indicated six genera**,** *Streptococcus*, *Peptostreptococcus*, *Selenomonas*, *Pseudomonas*, *Prevotella*, and *Fusobacterium*, may have potential diagnostic biomarkers for distinguishing GC from NGC. Interestingly, all these genera belong to oral microorganisms, which suggests that oral flora play important roles in the development of GC. *Streptococcus* is a common purulent Gram-positive coccus widely present in the human gastrointestinal tract and nasopharynx. Its elevation was seen in multiple types of samples from various gastrointestinal diseases, and its view as a pathogenic microorganism is widely accepted (33). *Selenomonas*, a Gram-negative genus, can be found in the oral cavity, stomach, and feces and is associated with colon cancer (34). *Pseudomonas*, a Gram-negative aerobic bacterium, is a common conditional pathogenic bacteria. It has been reported that the abundance of *Pseudomonas* in serum is high in both GC and normal groups (35). *Prevotella*, a genus of Gram-negative anaerobic bacteria, is found in multiple sites in the human body, including the oral cavity and gastrointestinal tract (36). *Prevotella* was enriched in GC tissue (7, 9, 25) and decreased in saliva (37), suggesting that translocation of *Prevotella* from the oral cavity to the gastrointestinal tract is a cause of gastric disease development. It has been suggested that *Fusobacterium* is present in the infected gastrointestinal tract (38), and this may stem from the selective translocation of *Fusobacterium* in the oral cavity (39). *Fusobacterium* has also been demonstrated to be associated with IBD and colorectal cancer (CRC) (23, 40, 41). *Peptostreptococcus* belongs to a group of Gram-positive anaerobic bacteria present in the gastrointestinal tract and vagina. Coker et al. identified *Peptostreptococcus* as an important genus for gastritis to GC development (9). The above results indicated that the six differential genera are closely associated with gastric diseases and have potential as microbial markers for GC diagnosis.

Next, we explored the possibility of each of these six genera alone as diagnostic markers for GC. The diagnostic value of *Streptococcus* performed well in some samples, but not others, as did the other five genera. Studies on *Streptococcus* as a diagnostic marker have been reported, for example, Zhou et al. diagnosed GC by detecting *Streptococcus anginosus* and *Streptococcus constellatus* in stool samples with AUC of 0.91 (10). Yu et al (33). explored *Streptococcus* as a marker of liver metastasis in GC with an AUC of 0.651. *Streptococcus* has also been reported to be significantly elevated in CRC (42, 43), suggesting its potency as a diagnostic marker for gastrointestinal cancer. However, our meta-analysis did not verify the above results. We speculated that the reason may be that the cases in our study were from gastric mucosal tissue rather than fecal samples. This suggests that *Streptococcus* may have poor tissue specificity and diagnostic efficacy as a marker, so its feasibility as a single diagnostic marker for GC remains to be explored. The other five genera have been combined as diagnostic markers in previous studies (32, 37, 44), but no studies have investigated them as markers alone. Our study showed that these genera suffered from poor diagnostic efficacy when used alone as diagnostic markers for GC.

To find the marker combinations that performed best in the 16S RNA data of gastric mucosa, we constructed a random forest model in each of the Matched group data, and progressively added genera based on the order of importance to obtain six marker combinations, of which the combination consisting of five genera (*Streptococcus*, *Peptostreptococcus*, *Selenomonas*, *Pseudomonas*, and *Prevotella*) had the best diagnostic power with a median AUC value of 0.7525 (range: 0.5859–0.9350). In exploring the best combination of biomarkers, we found that the performance of each combination was excellent in the S3 data set, with AUC values of all the COMs signatures equal to 0.9. After analyzing the expression and diagnostic ability of single microbial genera, we believe that this may be due to two reasons. First, in S3, the expression levels of *Streptococcus*, *Peptostreptococcus*, and *Selenomonas* in GC were significantly higher than those in NGC, and the differences were large. Second, *Streptococcus*, *Peptostreptococcus*, and *Selenomonas* all had excellent diagnostic efficiency as single microbial genera in S3. Furthermore, we found that the COM5 had good diagnostic ability for the population in Hohhot, Inner Mongolia (S3 AUC = 0.935, S10 AUC = 0.934), which suggests that developing region-specific diagnostic biomarkers to improve diagnostic ability may be feasible. Then, the diagnostic value of this combination was verified with mucosal samples from the Unmatched group, and tongue, oral wipe, stool, and gastric fluid samples in Other group, and the median AUC value was up to 0.8818 (range: 0.7397–0.9533). However, there have been no reports using the combination of these five genera for GC diagnosis. These results indicated that this combination of genera has good diagnostic efficacy and wide applicability for patients with GC, which may open a door for the non-invasive diagnosis of GC.

In addition, there are some shortcomings in this study (2). The effect of *Hp* on gastric microbial community composition has been demonstrated (45), but in this study, it was not possible to subgroup *Hp* for a more precise analysis because of incomplete *Hp* data (3). The number of studies included in this meta-analysis was still small, and some did not have a large sample size, which may affect some of the results (4). There were insufficient data for other types of samples, such as oral and fecal samples with only one or two datasets each, giving insufficient validation strength. In the future, more informative and comprehensive studies are needed to verify the microbial diagnostic markers of GC obtained in this study.

In conclusion, leveraging existing 16S rRNA microbial data, we demonstrated the significant differences in gastric microbiota between GC and non-cancerous patients and obtained a combination of genera *Streptococcus*, *Peptostreptococcus*, *Selenomonas*, *Pseudomonas,* and *Prevotella* had excellent performance in screening GC with broad applicability and good diagnostic efficacy for the Chinese population. Our results would lend support to the use of microbial markers in detecting GC. Moreover, these biomarkers might also be plausible candidates for further mechanistic research into the role of the microbiota in tumorigenesis.

## SUPPLEMENTAL MATERIAL

Fig. S1 msystems.00747-23-s0001.pdf The alpha diversity indicators and microbial composition of each data set in the Other group.

Fig. S2 msystems.00747-23-s0002.pdf The relative abundance of 6 genera in 22 data sets, NGC and GC groups.

Fig. S3 msystems.00747-23-s0003.pdf AUC values of *Peptostreptococcus*, *Selenomonas*, *Pseudomonas*, *Prevotella*, and *Fusobacterium* in each data set.

Supplemental Legends msystems.00747-23-s0004.docx Supplemental figure and table legends.

Table S1 msystems.00747-23-s0005.xlsx Alpha diversity and microbiota composition of studies included in the matched group.

Table S2 msystems.00747-23-s0006.xlsx Alpha diversity and microbiota composition of studies included in the unmatched group.

Table S3 msystems.00747-23-s0007.xlsx Alpha diversity and microbiota composition of studies included in the other group.

Table S4 msystems.00747-23-s0008.xlsx Comparison of community dissimilarity between individuals with GCs and NGCs as tested using PERMANOVA.

ASM does not own the copyrights to Supplemental Material that may be linked to, or accessed through, an article. The authors have granted ASM a non-exclusive, world-wide license to publish the Supplemental Material files. Please contact the corresponding author directly for reuse.

## References

[1] Sung H , Ferlay J , Siegel RL , Laversanne M , Soerjomataram I , Jemal A , Bray F . 2021. Global Cancer Statistics 2020: GLOBOCAN estimates of incidence and mortality worldwide for 36 cancers in 185 countries. CA Cancer J Clin 71:209–249. doi:10.3322/caac.21660 33538338

[2] Ferlay J , Ervik M , Lam F , Colombet M , Mery L , Piñeros M , Znaor A , Soerjomataram I , Bray F . 2018. Global cancer observatory: cancer today. Lyon, France International agency for research on cancer

[3] Cao W , Chen HD , Yu YW , Li N , Chen WQ . 2021. Changing profiles of cancer burden worldwide and in China: a secondary analysis of the global cancer statistics 2020. Chin Med J (Engl) 134:783–791. doi:10.1097/CM9.0000000000001474 33734139 PMC8104205

[4] Kauppila JH , Selander KS . 2014. Toll-like receptors in esophageal cancer. Front Immunol 5:200. doi:10.3389/fimmu.2014.00200 24847326 PMC4019875

[5] Yang L , Chaudhary N , Baghdadi J , Pei Z . 2014. Microbiome in reflux disorders and esophageal adenocarcinoma. Cancer J 20:207–210. doi:10.1097/PPO.0000000000000044 24855009 PMC4120752

[6] Chen X-H , Wang A , Chu A-N , Gong Y-H , Yuan Y . 2019. Mucosa-associated microbiota in gastric cancer tissues compared with non-cancer tissues. Front Microbiol 10:1261. doi:10.3389/fmicb.2019.01261 31231345 PMC6560205

[7] Dai D , Yang Y , Yu J , Dang T , Qin W , Teng L , Ye J , Jiang H . 2021. Interactions between gastric microbiota and metabolites in gastric cancer. Cell Death Dis 12:1104. doi:10.1038/s41419-021-04396-y 34819503 PMC8613192

[8] Liu W , Zhang J , Wu C , Cai S , Huang W , Chen J , Xi X , Liang Z , Hou Q , Zhou B , Qin N , Zhang H . 2016. Unique features of ethnic Mongolian gut microbiome revealed by metagenomic analysis. Sci Rep 6:34826. doi:10.1038/srep34826 27708392 PMC5052615

[9] Coker OO , Dai Z , Nie Y , Zhao G , Cao L , Nakatsu G , Wu WK , Wong SH , Chen Z , Sung JJY , Yu J . 2018. Mucosal microbiome dysbiosis in gastric carcinogenesis. Gut 67:1024–1032. doi:10.1136/gutjnl-2017-314281 28765474 PMC5969346

[10] Zhou C-B , Pan S-Y , Jin P , Deng J-W , Xue J-H , Ma X-Y , Xie Y-H , Cao H , Liu Q , Xie W-F , Zou X-P , Sheng J-Q , Wang B-M , Wang H , Ren J-L , Liu S-D , Sun Y-W , Meng X-J , Zhao G , Chen J-X , Cui Y , Wang P-Q , Guo H-M , Yang L , Chen X , Ding J , Yang X-N , Wang X-K , Qian A-H , Hou L-D , Wang Z , Chen Y-X , Fang J-Y . 2022. Fecal signatures of Streptococcus anginosus and Streptococcus constellatus for noninvasive screening and early warning of gastric cancer. Gastroenterology 162:1933–1947. doi:10.1053/j.gastro.2022.02.015 35167866

[11] Bolyen E , Rideout JR , Dillon MR , Bokulich NA , Abnet CC , Al-Ghalith GA , Alexander H , Alm EJ , Arumugam M , Asnicar F , Bai Y , Bisanz JE , Bittinger K , Brejnrod A , Brislawn CJ , Brown CT , Callahan BJ , Caraballo-Rodríguez AM , Chase J , Cope EK , Da Silva R , Diener C , Dorrestein PC , Douglas GM , Durall DM , Duvallet C , Edwardson CF , Ernst M , Estaki M , Fouquier J , Gauglitz JM , Gibbons SM , Gibson DL , Gonzalez A , Gorlick K , Guo J , Hillmann B , Holmes S , Holste H , Huttenhower C , Huttley GA , Janssen S , Jarmusch AK , Jiang L , Kaehler BD , Kang KB , Keefe CR , Keim P , Kelley ST , Knights D , Koester I , Kosciolek T , Kreps J , Langille MGI , Lee J , Ley R , Liu Y-X , Loftfield E , Lozupone C , Maher M , Marotz C , Martin BD , McDonald D , McIver LJ , Melnik AV , Metcalf JL , Morgan SC , Morton JT , Naimey AT , Navas-Molina JA , Nothias LF , Orchanian SB , Pearson T , Peoples SL , Petras D , Preuss ML , Pruesse E , Rasmussen LB , Rivers A , Robeson MS , Rosenthal P , Segata N , Shaffer M , Shiffer A , Sinha R , Song SJ , Spear JR , Swafford AD , Thompson LR , Torres PJ , Trinh P , Tripathi A , Turnbaugh PJ , Ul-Hasan S , van der Hooft JJJ , Vargas F , Vázquez-Baeza Y , Vogtmann E , von Hippel M , Walters W , Wan Y , Wang M , Warren J , Weber KC , Williamson CHD , Willis AD , Xu ZZ , Zaneveld JR , Zhang Y , Zhu Q , Knight R , Caporaso JG . 2019. Reproducible, interactive, scalable and extensible microbiome data science using QIIME 2. Nat Biotechnol 37:852–857. doi:10.1038/s41587-019-0252-6 31341288 PMC7015180

[12] Rognes T , Flouri T , Nichols B , Quince C , Mahé F . 2016. VSEARCH: a versatile open source tool for metagenomics. PeerJ 4:e2584. doi:10.7717/peerj.2584 27781170 PMC5075697

[13] Amir A , McDonald D , Navas-Molina JA , Kopylova E , Morton JT , Zech Xu Z , Kightley EP , Thompson LR , Hyde ER , Gonzalez A , Knight R . 2017. Deblur rapidly resolves single-nucleotide community sequence patterns. mSystems 2:e00191-16. doi:10.1128/mSystems.00191-16 28289731 PMC5340863

[14] Bokulich NA , Kaehler BD , Rideout JR , Dillon M , Bolyen E , Knight R , Huttley GA , Gregory Caporaso J . 2018. Optimizing taxonomic classification of marker-gene amplicon sequences with QIIME 2's q2-feature-classifier plugin. Microbiome 6:90. doi:10.1186/s40168-018-0470-z 29773078 PMC5956843

[15] Segata N , Izard J , Waldron L , Gevers D , Miropolsky L , Garrett WS , Huttenhower C . 2011. Metagenomic biomarker discovery and explanation. Genome Biol 12:R60. doi:10.1186/gb-2011-12-6-r60 21702898 PMC3218848

[16] Liaw A , Wiener M . 2002. Classification and Regression by RandomForest. R News

[17] Tseng C-H , Lin J-T , Ho HJ , Lai Z-L , Wang C-B , Tang S-L , Wu C-Y . 2016. Gastric microbiota and predicted gene functions are altered after subtotal gastrectomy in patients with gastric cancer. Sci Rep 6:20701. doi:10.1038/srep20701 26860194 PMC4748256

[18] Yu G , Torres J , Hu N , Medrano-Guzman R , Herrera-Goepfert R , Humphrys MS , Wang L , Wang C , Ding T , Ravel J , Taylor PR , Abnet CC , Goldstein AM . 2017. Molecular characterization of the human stomach microbiota in gastric cancer patients. Front Cell Infect Microbiol 7:302. doi:10.3389/fcimb.2017.00302 28730144 PMC5498480

[19] Ling Z , Shao L , Liu X , Cheng Y , Yan C , Mei Y , Ji F , Liu X . 2019. Regulatory T cells and plasmacytoid dendritic cells within the tumor microenvironment in gastric cancer are correlated with gastric microbiota dysbiosis: a preliminary study. Front Immunol 10:533. doi:10.3389/fimmu.2019.00533 30936882 PMC6433099

[20] Godon JJ , Zumstein E , Dabert P , Habouzit F , Moletta R . 1997. Molecular microbial diversity of an anaerobic digestor as determined by small-subunit rDNA sequence analysis. Appl Environ Microbiol 63:2802–2813. doi:10.1128/aem.63.7.2802-2813.1997 9212428 PMC168577

[21] Zhang C , Hu A , Li J , Zhang F , Zhong P , Li Y , Li Y . 2022. Combined non-invasive prediction and new biomarkers of oral and fecal microbiota in patients with gastric and colorectal cancer. Front Cell Infect Microbiol 12:830684. doi:10.3389/fcimb.2022.830684 35663463 PMC9161364

[22] He C , Peng C , Shu X , Wang H , Zhu Z , Ouyang Y , Yang X , Xie C , Hu Y , Li N , Ge Z , Zhu Y , Lu N . 2022. Convergent dysbiosis of gastric mucosa and fluid microbiome during stomach carcinogenesis. Gastric Cancer 25:837–849. doi:10.1007/s10120-022-01302-z 35661945

[23] Feng Q , Liang S , Jia H , Stadlmayr A , Tang L , Lan Z , Zhang D , Xia H , Xu X , Jie Z , Su L , Li X , Li X , Li J , Xiao L , Huber-Schönauer U , Niederseer D , Xu X , Al-Aama JY , Yang H , Wang J , Kristiansen K , Arumugam M , Tilg H , Datz C , Wang J . 2015. Gut microbiome development along the colorectal adenoma-carcinoma sequence. Nat Commun 6:6528. doi:10.1038/ncomms7528 25758642

[24] Wang Z , Gao X , Zeng R , Wu Q , Sun H , Wu W , Zhang X , Sun G , Yan B , Wu L , Ren R , Guo M , Peng L , Yang Y . 2020. Changes of the gastric mucosal microbiome associated with histological stages of gastric carcinogenesis. Front Microbiol 11:997. doi:10.3389/fmicb.2020.00997 32547510 PMC7272699

[25] Zhang X , Li C , Cao W , Zhang Z . 2021. Alterations of gastric microbiota in gastric cancer and precancerous stages. Front Cell Infect Microbiol 11:559148. doi:10.3389/fcimb.2021.559148 33747975 PMC7966516

[26] Li F , Zhu H , Tao K , Xia Y , Liu M , Wang Y , Sun Y , Cao T , Chai J , Ni F , Shi B , Xu H . 2021. Mucosal microbial microenvironment in early gastric neoplasia and non-neoplastic gastric disease. J Gastroenterol Hepatol 36:3092–3101. doi:10.1111/jgh.15565 34089623

[27] Qi Y-F , Sun J-N , Ren L-F , Cao X-L , Dong J-H , Tao K , Guan X-M , Cui Y-N , Su W . 2019. Intestinal microbiota is altered in patients with gastric cancer from Shanxi province, China. Dig Dis Sci 64:1193–1203. doi:10.1007/s10620-018-5411-y 30535886

[28] Liu X , Shao L , Liu X , Ji F , Mei Y , Cheng Y , Liu F , Yan C , Li L , Ling Z . 2019. Alterations of gastric mucosal microbiota across different stomach microhabitats in a cohort of 276 patients with gastric cancer. EBioMedicine 40:336–348. doi:10.1016/j.ebiom.2018.12.034 30584008 PMC6412016

[29] Liu AQ , Vogtmann E , Shao DT , Abnet CC , Dou HY , Qin Y , Su Z , Wei WQ , Chen W . 2019. A comparison of biopsy and mucosal SWAB specimens for examining the microbiota of upper gastrointestinal carcinoma. Cancer Epidemiol Biomarkers Prev 28:2030–2037. doi:10.1158/1055-9965.EPI-18-1210 31519703 PMC7294753

[30] Wang L , Zhou J , Xin Y , Geng C , Tian Z , Yu X , Dong Q . 2016. Bacterial overgrowth and diversification of microbiota in gastric cancer. Eur J Gastroenterol Hepatol 28:261–266. doi:10.1097/MEG.0000000000000542 26657453 PMC4739309

[31] Wang L , Xin Y , Zhou J , Tian Z , Liu C , Yu X , Meng X , Jiang W , Zhao S , Dong Q . 2020. Gastric mucosa-associated microbial signatures of early gastric cancer. Front Microbiol 11:1548. doi:10.3389/fmicb.2020.01548 32733423 PMC7358557

[32] Xu S , Xiang C , Wu J , Teng Y , Wu Z , Wang R , Lu B , Zhan Z , Wu H , Zhang J . 2021. Tongue coating bacteria as a potential stable biomarker for gastric cancer independent of lifestyle. Dig Dis Sci 66:2964–2980. doi:10.1007/s10620-020-06637-0 33044677

[33] Yu D , Yang J , Jin M , Zhou B , Shi L , Zhao L , Zhang J , Lin Z , Ren J , Liu L , Zhang T , Liu H . 2021. Fecal Streptococcus alteration is associated with gastric cancer occurrence and liver metastasis. mBio 12:e0299421. doi:10.1128/mBio.02994-21 34872346 PMC8649758

[34] Allali I , Boukhatem N , Bouguenouch L , Hardi H , Boudouaya HA , Cadenas MB , Ouldim K , Amzazi S , Azcarate-Peril MA , Ghazal H . 2018. Gut microbiome of Moroccan colorectal cancer patients. Med Microbiol Immunol 207:211–225. doi:10.1007/s00430-018-0542-5 29687353 PMC6096775

[35] Dong Z , Chen B , Pan H , Wang D , Liu M , Yang Y , Zou M , Yang J , Xiao K , Zhao R , Zheng X , Zhang L , Zhang Y . 2019. Detection of microbial 16S rRNA gene in the serum of patients with gastric cancer. Front Oncol 9:608. doi:10.3389/fonc.2019.00608 31338330 PMC6629868

[36] Tett A , Pasolli E , Masetti G , Ercolini D , Segata N . 2021. Prevotella diversity, niches and interactions with the human host. Nat Rev Microbiol 19:585–599. doi:10.1038/s41579-021-00559-y 34050328 PMC11290707

[37] Huang K , Gao X , Wu L , Yan B , Wang Z , Zhang X , Peng L , Yu J , Sun G , Yang Y . 2021. Salivary microbiota for gastric cancer prediction: an exploratory study. Front Cell Infect Microbiol 11:640309. doi:10.3389/fcimb.2021.640309 33777850 PMC7988213

[38] Swidsinski A , Dörffel Y , Loening-Baucke V , Theissig F , Rückert JC , Ismail M , Rau WA , Gaschler D , Weizenegger M , Kühn S , Schilling J , Dörffel WV . 2011. Acute appendicitis is characterised by local invasion with Fusobacterium nucleatum/necrophorum. Gut 60:34–40. doi:10.1136/gut.2009.191320 19926616

[39] Richardson M , Ren J , Rubinstein MR , Taylor JA , Friedman RA , Shen B , Han YW . 2020. Analysis of 16S rRNA genes reveals reduced Fusobacterial community diversity when translocating from saliva to GI sites. Gut Microbes 12:1–13. doi:10.1080/19490976.2020.1814120 PMC7577115 33054632

[40] Castellarin M , Warren RL , Freeman JD , Dreolini L , Krzywinski M , Strauss J , Barnes R , Watson P , Allen-Vercoe E , Moore RA , Holt RA . 2012. Fusobacterium nucleatum infection is prevalent in human colorectal carcinoma. Genome Res 22:299–306. doi:10.1101/gr.126516.111 22009989 PMC3266037

[41] Strauss J , Kaplan GG , Beck PL , Rioux K , Panaccione R , Devinney R , Lynch T , Allen-Vercoe E . 2011. Invasive potential of gut mucosa-derived Fusobacterium nucleatum positively correlates with IBD status of the host. Inflamm Bowel Dis 17:1971–1978. doi:10.1002/ibd.21606 21830275

[42] Flemer B , Warren RD , Barrett MP , Cisek K , Das A , Jeffery IB , Hurley E , O’Riordain M , Shanahan F , O’Toole PW . 2018. The oral microbiota in colorectal cancer is distinctive and predictive. Gut 67:1454–1463. doi:10.1136/gutjnl-2017-314814 28988196 PMC6204958

[43] Dinakaran V , Mandape SN , Shuba K , Pratap S , Sakhare SS , Tabatabai MA , Smoot DT , Farmer-Dixon CM , Kesavalu LN , Adunyah SE , Southerland JH , Gangula PR . 2018. Identification of specific oral and gut pathogens in full thickness colon of colitis patients: implications for colon motility. Front Microbiol 9:3220. doi:10.3389/fmicb.2018.03220 30666239 PMC6330997

[44] Xu J , Xiang C , Zhang C , Xu B , Wu J , Wang R , Yang Y , Shi L , Zhang J , Zhan Z . 2019. Microbial biomarkers of common tongue coatings in patients with gastric cancer. Microb Pathog 127:97–105. doi:10.1016/j.micpath.2018.11.051 30508628

[45] Wang D , Zhang T , Lu Y , Wang C , Wu Y , Li J , Tao Y , Deng L , Zhang X , Ma J . 2022. Helicobacter pylori infection affects the human gastric microbiome, as revealed by metagenomic sequencing. FEBS Open Bio 12:1188–1196. doi:10.1002/2211-5463.13390 PMC9157398 35243810

