# Supplementary figures and images for: Leveraging existing 16S rRNA microbial data to identify diagnostic biomarker in Chinese patients with gastric cancer: a systematic meta-analysis
Source: mSystems. 2023 Oct 3;8(5):e00747-23. doi: 10.1128/msystems.00747-23 (PMC10654077; doi:10.1128/msystems.00747-23)

A

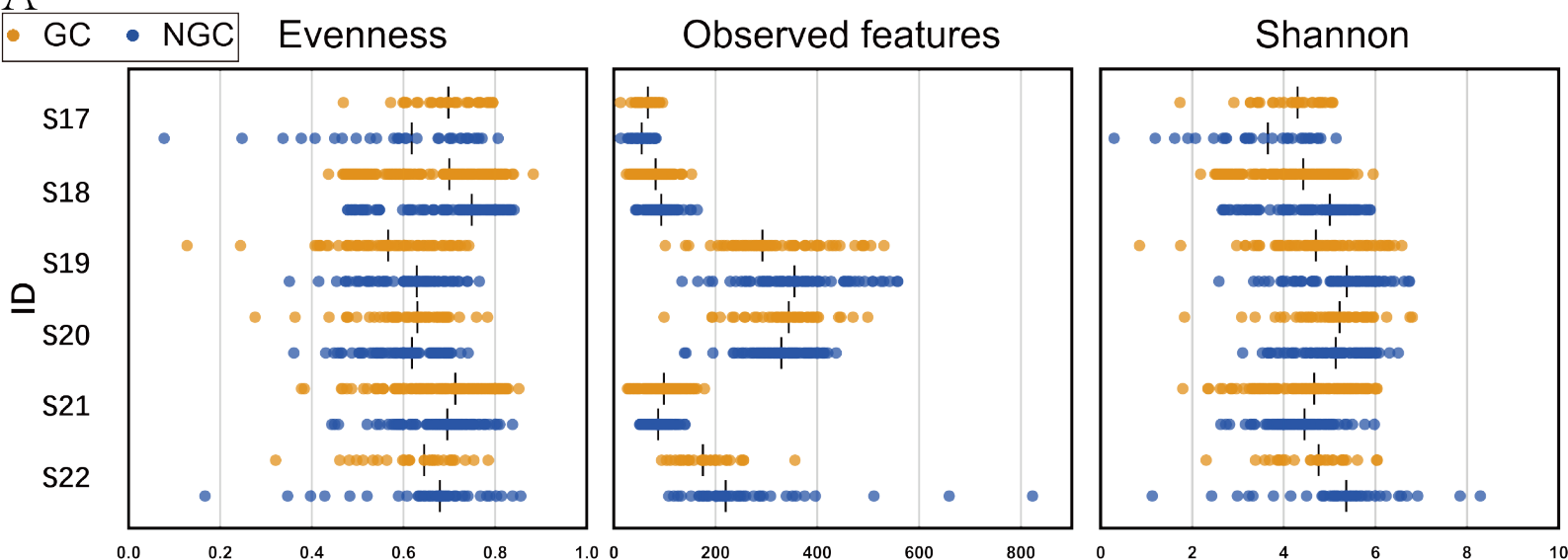

B

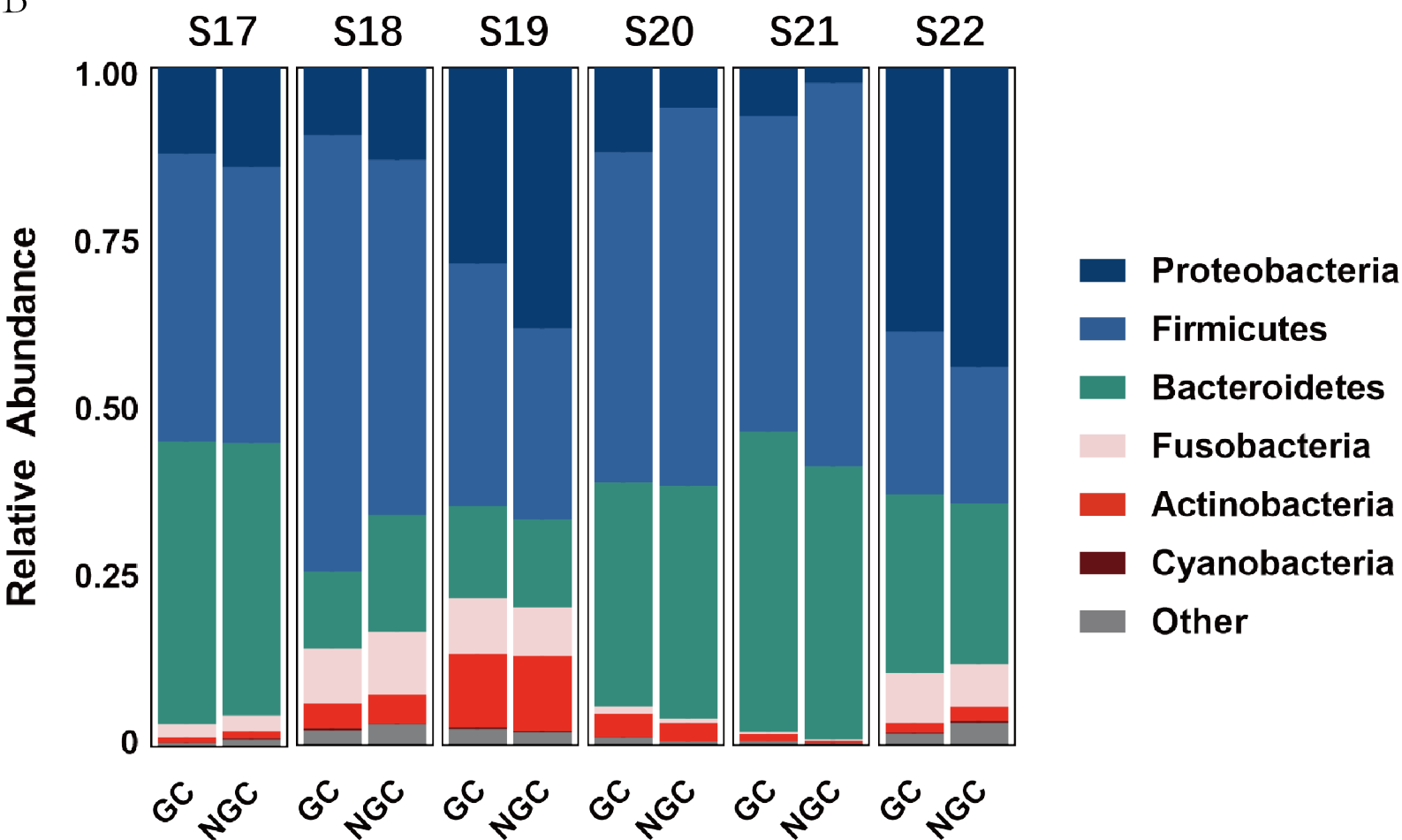

Supplement: Fig. S1 — The alpha diversity indicators and microbial composition of each data set in the Other group. [file msystems.00747-23-s0001.pdf]

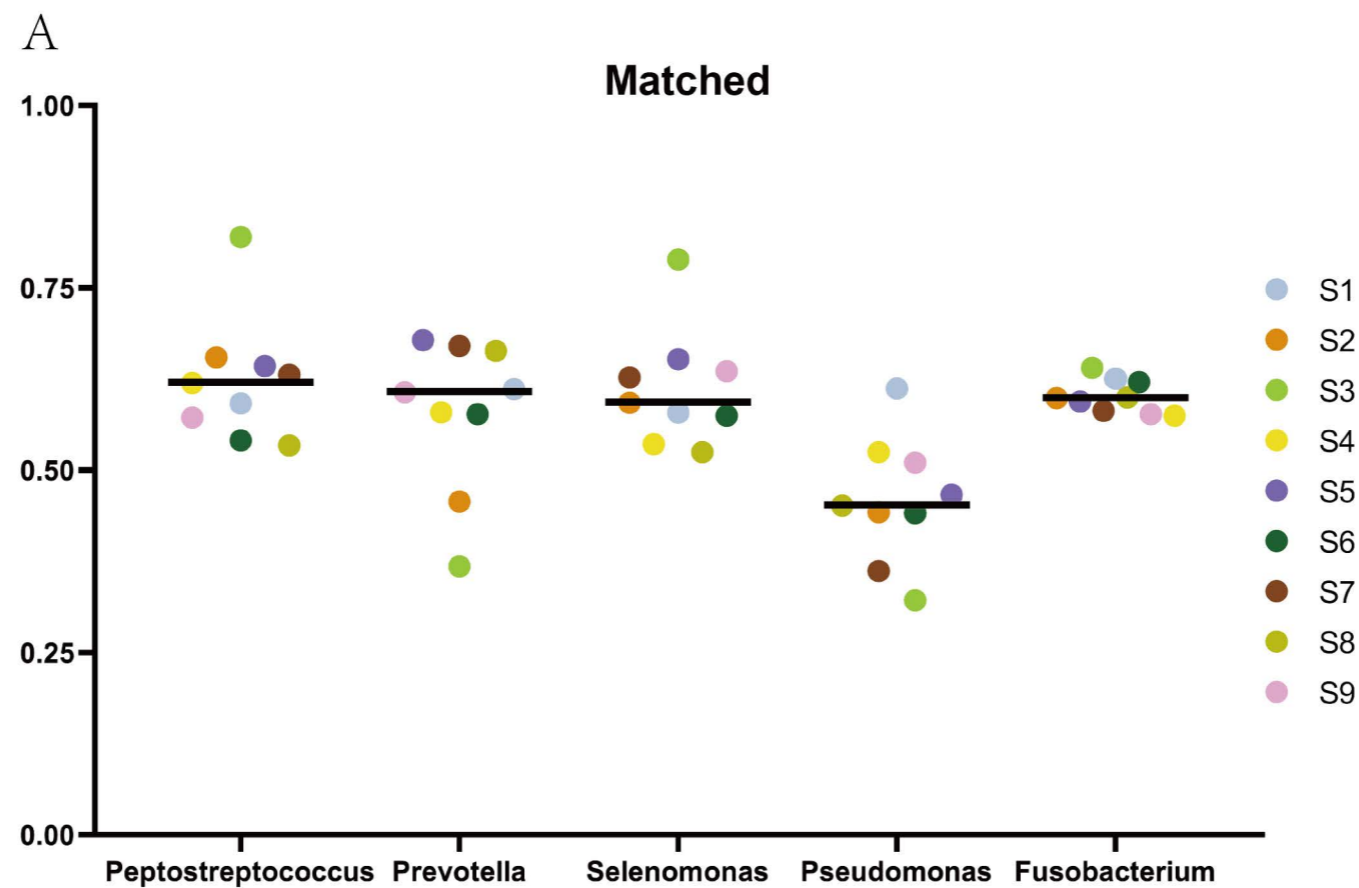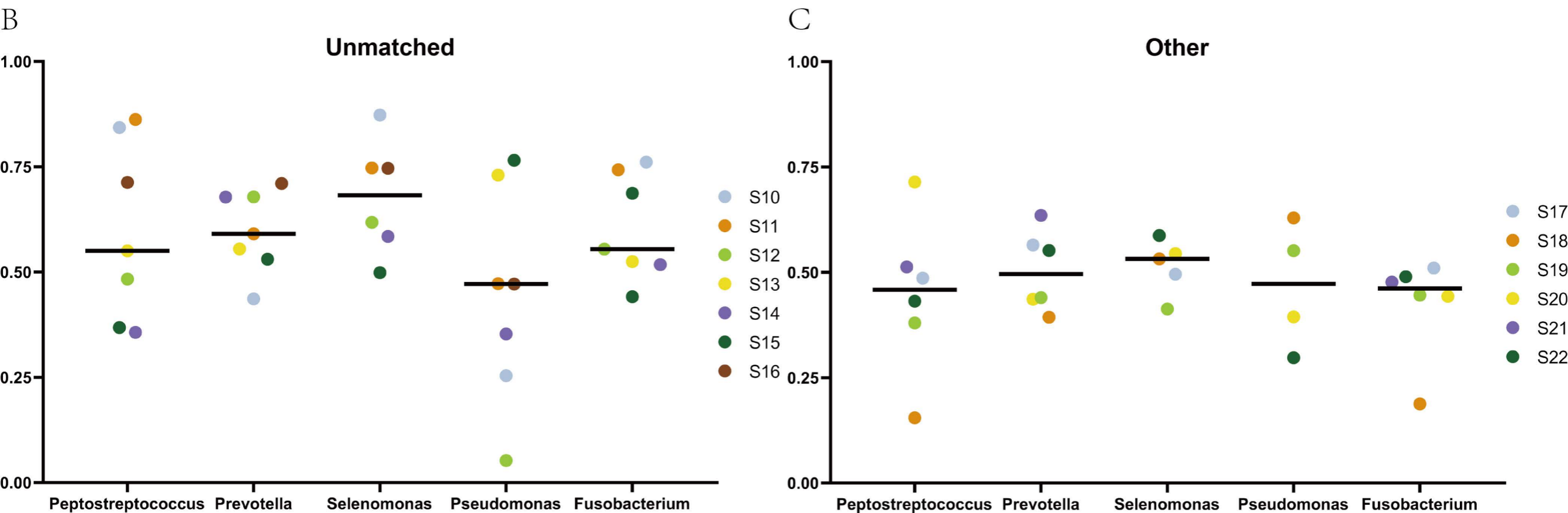

Supplement: Fig. S3 — AUC values of Peptostreptococcus, Selenomonas, Pseudomonas, Prevotella, and Fusobacterium in each data set. [file msystems.00747-23-s0003.pdf]
