# Supplementary material for: Leveraging existing 16S rRNA microbial data to identify diagnostic biomarker in Chinese patients with gastric cancer: a systematic meta-analysis
Source: mSystems. 2023 Oct 3;8(5):e00747-23. doi: 10.1128/msystems.00747-23 (PMC10654077; doi:10.1128/msystems.00747-23)
Supplement: Supplemental Legends — Supplemental figure and table legends. [file msystems.00747-23-s0004.docx]

**Supplemental Figure Legends**

Fig. S1. The alpha diversity indicators and microbial composition of each data set in the Other group. (A) Alpha diversity indicators evenness index, observed features, and Shannon index in the Other group of data in each group. (B) The microbiota composition of each data set in the Other group.

Fig. S2. The relative abundance of 6 genera in 22 datasets NGC and GC groups. (A) *Streptococcus*. (B) *Peptostreptococcus*. (C) *Selenomonas*. (D) *Pseudomonas*. (E) *Prevotella*. (F) *Fusobacterium*.

Fig. S3. AUC values of *Peptostreptococcus*, *Selenomonas*, *Pseudomonas*, *Prevotella*, and *Fusobacterium* in each data set. (A) AUC values of the five genera in the Matched group for each data set. (B) AUC values of the five genera in the Unmatched group for each data set. (C) AUC values of the five genera in the Other group for each data set.

**Supplemental Table Legends**

Table S1. Alpha diversity and microbiota composition of studies included in the matched group.

Table S2. Alpha diversity and microbiota composition of studies included in the unmatched group.

Table S3. Alpha diversity and microbiota composition of studies included in the other group.

Table S4. Comparison of community dissimilarity between individuals with GCs and NGCs as tested using PERMANOVA
