# Supplementary material for: Leveraging existing 16S rRNA microbial data to identify diagnostic biomarker in Chinese patients with gastric cancer: a systematic meta-analysis
Source: mSystems. 2023 Oct 3;8(5):e00747-23. doi: 10.1128/msystems.00747-23 (PMC10654077; doi:10.1128/msystems.00747-23)
Supplement: Fig. S2 — The relative abundance of 6 genera in 22 data sets, NGC and GC groups. [file msystems.00747-23-s0002.pdf]

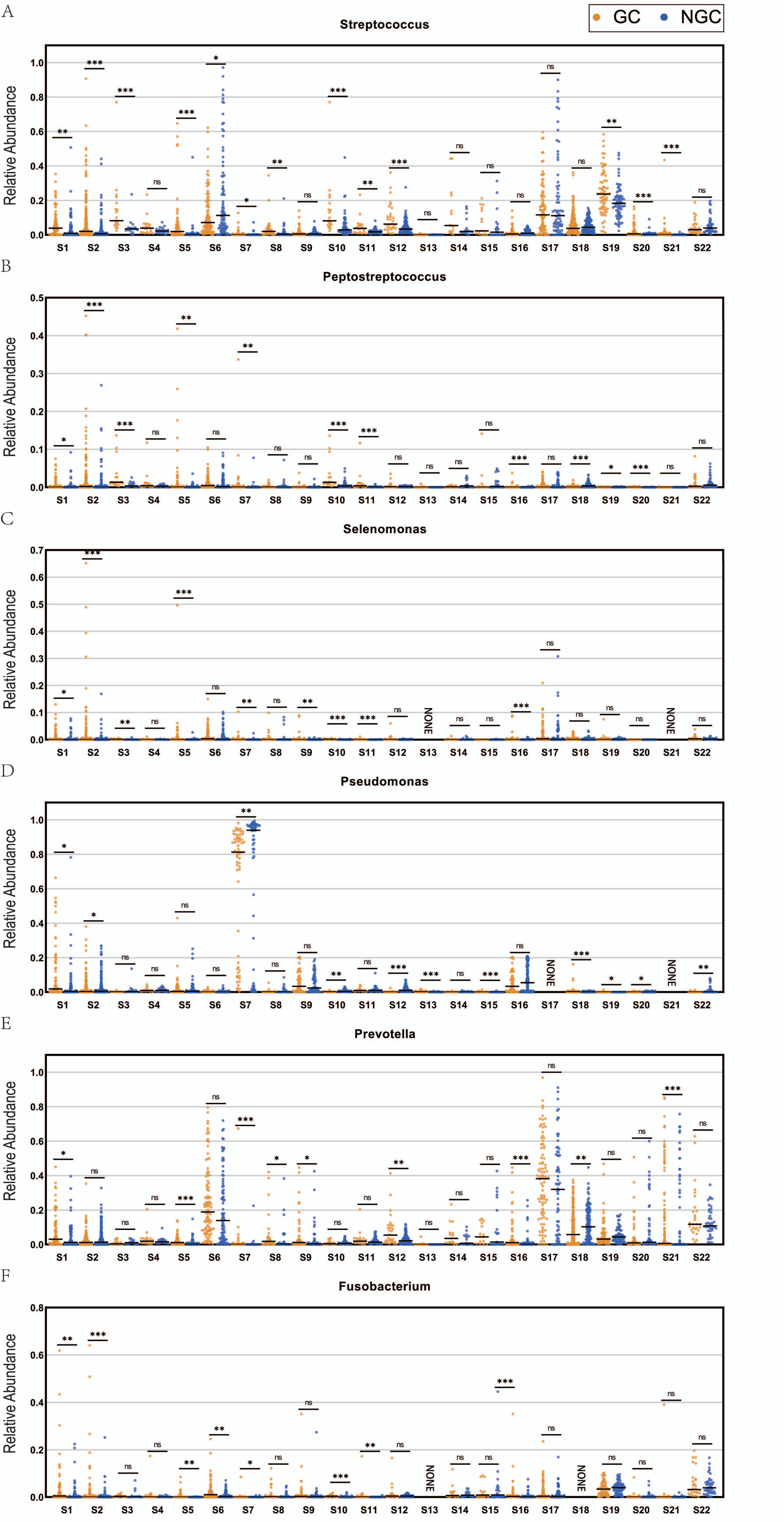
GC
NGC
A
Streptococcus
Relative Abundance
0.0 0.2 0.4 0.6 0.8 1.0
** *** *** ns *** * * ** ns *** ** *** ns ns ns ns ns ns ** *** *** ns
S1 S2 S3 S4 S5 S6 S7 S8 S9 S10 S11 S12 S13 S14 S15 S16 S17 S18 S19 S20 S21 S22
B
Peptostreptococcus
Relative Abundance
0.0 0.1 0.2 0.3 0.4 0.5
* *** *** ns ** ns ** ns ns *** *** ns ns ns ns *** ns *** * *** ns ns
S1 S2 S3 S4 S5 S6 S7 S8 S9 S10 S11 S12 S13 S14 S15 S16 S17 S18 S19 S20 S21 S22
C
Selenomonas
Relative Abundance
0.0 0.1 0.2 0.3 0.4 0.5 0.6 0.7
* *** ** ns *** ns ** ns ** *** *** ns NONE ns ns *** ns ns ns ns NONE ns
S1 S2 S3 S4 S5 S6 S7 S8 S9 S10 S11 S12 S13 S14 S15 S16 S17 S18 S19 S20 S21 S22
D
Pseudomonas
Relative Abundance
0.0 0.2 0.4 0.6 0.8 1.0
* * ns ns ns ns ** ns ns ** ns *** *** ns *** ns NONE *** * * NONE **
S1 S2 S3 S4 S5 S6 S7 S8 S9 S10 S11 S12 S13 S14 S15 S16 S17 S18 S19 S20 S21 S22
E
Prevotella
Relative Abundance
0.0 0.2 0.4 0.6 0.8 1.0
* ns ns ns *** ns *** * * ns ns ** ns ns ns *** ns ** ns ns *** ns
S1 S2 S3 S4 S5 S6 S7 S8 S9 S10 S11 S12 S13 S14 S15 S16 S17 S18 S19 S20 S21 S22
F
Fusobacterium
Relative Abundance
0.0 0.2 0.4 0.6 0.8
** *** ns ns ** ** * ns ns *** ** ns NONE ns ns *** ns NONE ns ns ns ns
S1 S2 S3 S4 S5 S6 S7 S8 S9 S10 S11 S12 S13 S14 S15 S16 S17 S18 S19 S20 S21 S22
